# Relationship between Neuronal Damage/Death and Astrogliosis in the Cerebral Motor Cortex of Gerbil Models of Mild and Severe Ischemia and Reperfusion Injury

**DOI:** 10.3390/ijms23095096

**Published:** 2022-05-03

**Authors:** Choong-Hyun Lee, Tae-Kyeong Lee, Dae Won Kim, Soon Sung Lim, Il Jun Kang, Ji Hyeon Ahn, Joon Ha Park, Jae-Chul Lee, Choong-Hyo Kim, Yoonsoo Park, Moo-Ho Won, Soo Young Choi

**Affiliations:** 1 Department of Pharmacy, College of Pharmacy, Dankook University, Cheonan 31116, Korea; anaphy@dankook.ac.kr; 2 Department of Food Science and Nutrition, Hallym University, Chuncheon 24252, Korea; tk_lee@hallym.ac.kr (T.-K.L.); limss@hallym.ac.kr (S.S.L.); ijkang@hallym.ac.kr (I.J.K.); 3 Department of Biochemistry and Molecular Biology, Research Institute of Oral Sciences, College of Dentistry, Gangnung-Wonju National University, Gangneung 25457, Korea; kimdw@gwnu.ac.kr; 4 Department of Physical Therapy, College of Health Science, Youngsan University, Yangsan 50510, Korea; jh-ahn@ysu.ac.kr; 5 Department of Anatomy, College of Korean Medicine, Dongguk University, Gyeongju 38066, Korea; jh-park@dongguk.ac.kr; 6 Department of Neurobiology, School of Medicine, Kangwon National University, Chuncheon 24341, Korea; anajclee@kangwon.ac.kr; 7 Department of Neurosurgery, Kangwon National University Hospital, School of Medicine, Kangwon National University, Chuncheon 24289, Korea; jeuel@kangwon.ac.kr; 8 Department of Emergency Medicine, Kangwon National University Hospital, School of Medicine, Kangwon National University, Chuncheon 24289, Korea; pyoonsoo@naver.com; 9 Department of Biomedical Science, Research Institute for Bioscience and Biotechnology, Hallym University, Chuncheon 24252, Korea

**Keywords:** astrocyte endfeet, blood–brain barrier, cortical layer, glial fibrillary acidic protein, ischemia and reperfusion injury, S100

## Abstract

Neuronal loss (death) occurs selectively in vulnerable brain regions after ischemic insults. Astrogliosis is accompanied by neuronal death. It can change the molecular expression and morphology of astrocytes following ischemic insults. However, little is known about cerebral ischemia and reperfusion injury that can variously lead to damage of astrocytes according to the degree of ischemic injury, which is related to neuronal damage/death. Thus, the purpose of this study was to examine the relationship between damage to cortical neurons and astrocytes using gerbil models of mild and severe transient forebrain ischemia induced by blocking the blood supply to the forebrain for five or 15 min. Significant ischemia tFI-induced neuronal death occurred in the deep layers (layers V and VI) of the motor cortex: neuronal death occurred earlier and more severely in gerbils with severe ischemia than in gerbils with mild ischemia. Distinct astrogliosis was detected in layers V and VI. It gradually increased with time after both ischemiae. The astrogliosis was significantly higher in severe ischemia than in mild ischemia. The ischemia-induced increase of glial fibrillary acidic protein (GFAP; a maker of astrocyte) expression in severe ischemia was significantly higher than that in mild ischemia. However, GFAP-immunoreactive astrocytes were apparently damaged two days after both ischemiae. At five days after ischemiae, astrocyte endfeet around capillary endothelial cells were severely ruptured. They were more severely ruptured by severe ischemia than by mild ischemia. However, the number of astrocytes stained with S100 was significantly higher in severe ischemia than in mild ischemia. These results indicate that the degree of astrogliosis, including the disruption (loss) of astrocyte endfeet following ischemia and reperfusion in the forebrain, might depend on the severity of ischemia and that the degree of ischemia-induced neuronal damage may be associated with the degree of astrogliosis.

## 1. Introduction

Cerebral ischemic insults can lead to irreversible brain damage, loss of neuronal functions and neurologic impairments [1,2,3]. Especially, a temporary hindrance of blood supply in the brain triggers ischemia and reperfusion (IR) injury and it leads to a selective pattern of neurodegeneration in neurons (i.e., pyramidal neurons in the hippocampus and cerebral cortex) for a few days (2–5 days) after IR injury [4,5,6,7,8]. Many factors, such as age, sex, ischemic duration and brain/body temperature, can affect the pattern and degree of ischemic damage [9,10,11]. Among these factors, ischemic duration has been thought to be one of the major factors leading to ischemic injury, including neuronal damage, gliosis (astrocytosis and microgliosis) and blood–brain barrier (BBB) damage [12,13]. It has been studied on the patterns of neuronal death in the hippocampus following 5 min (mild), 15 min (severe) and 20 min (lethal) of ischemic duration in gerbils [13]. In this study, mild IR injury brings neuronal death in the hippocampal cornu ammonis (CA) 1 area; severe IR leads to neuronal death not only in the CA1-3 areas but also in the dentate gyrus, and animals subjected to lethal IR injury show high mortality [13].

It is well known that astrocytes are reactive to various central nervous system (CNS) damages (i.e., trauma, infection, stroke, autoimmune responses or neurodegenerative disease) called astrogliosis or reactive astrogliosis [13,14,15,16]. Astrogliosis can change the morphology and molecular expression of astrocytes in response to damage or infection of the CNS [16,17]. In healthy neural tissues, astrocytes play diverse and critical roles in the maintenance of ion homeostasis, neurotransmitter recycling, synapse formation and blood–brain barrier (BBB) function [18,19,20]. It has been generally accepted that cerebral ischemic insults can lead to astrogliosis, showing that a more serious ischemic injury can develop much higher astrogliosis [13,20,21]. Recently, it has been reported that IR injury can lead to damage/dysfunction of astrocytes, and it is closely associated with IR-induced neuronal damage or death (loss) [21,22,23].

Many previous studies have reported neuronal damage or death in the cerebral cortex using various animal models of cerebral ischemia [4,5,7,24,25]. However, cerebral IR-induced neuronal death/damage and its related mechanisms in the cerebral cortex have not been fully elucidated yet. Therefore, the objective of this study was to examine the relationship between IR-induced neuronal damage or death and astrogliosis, which is closely related to BBB disruption in the cerebral motor cortex of gerbil, as one suitable model for studying transient forebrain ischemia (tFI) to investigate the relationship between neuronal damage/death and astrogliosis. Gerbil models of mild and severe tFI were used.

## 2. Results

### 2.1. Change in Motor Behavior

**Figure 1:**
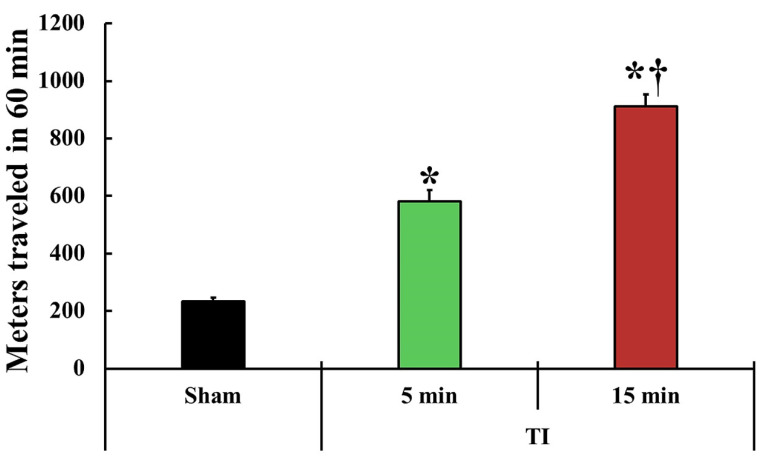
Change in spontaneous motor activity (SAM). SMA is evaluated in the traveled entire distance (meters) in 60 min at one day after tFI (*n* = 5 or 7, respectively; * *p* < 0.05, significantly different from the mild and severe sham group, and † *p* < 0.05, significantly different from the mild tFI group at the corresponding time). The bars indicate the means ± SEM.

To examine the tFI-induced change in motor behavior, spontaneous motor activity (SMA) was examined on day 1 after tFI in all groups (Figure 1). SMA (581 ± 38 m) in the mild tFI group was significantly higher (2.5-fold) than that (233 ± 13 m) in the mild sham group. SMA (910 ± 43 m) in the severe tFI group was significantly increased: it was 3.9-fold as compared to the sham group and 1.6-fold when compared to the mild tFI group.

### 2.2. Neuronal Damage/Death (Loss)

#### 2.2.1. Cresyl Violet (CV)-Stained Cells (CV-Cells)

**Figure 2:**
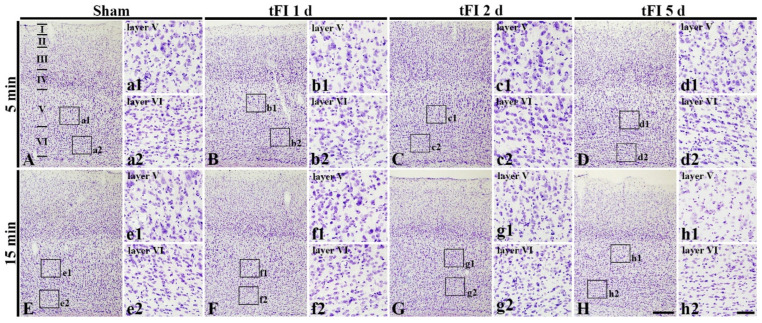
CV staining in the motor cortex of mild sham (**A**,**a1**,**a2**), mild tFI (**B**–**D**,**b1**–**d1**,**b2**–**d2**), severe sham (**E**,**e1**,**e2**), and severe tFI (**F**–**H**,**f1**–**h2**,**f2**–**h2**) groups at one day (**B**,**b1**,**b2**,**F**,**f1**,**f2**), 2 (**C**,**c1**,**c2**, **G**,**g1**,**g2**) and five days (**D**,**d1**,**d2**,**H**,**h1**,**h2**) after tFI. At five days after tFI, CV-cells are apparently decreased and damaged in layers V and VI (**d1**,**d2**,**h1**,**h2**) in both of the tFI groups: more reduction and damage of CV-cells are shown in the severe tFI group (**h1**,**h2**) than the mild tFI group. Scale bars = 200 μm (**A**–**H**) and 50 μm (**a1**–**h1**,**a2**–**h2**).

**Figure 3:**
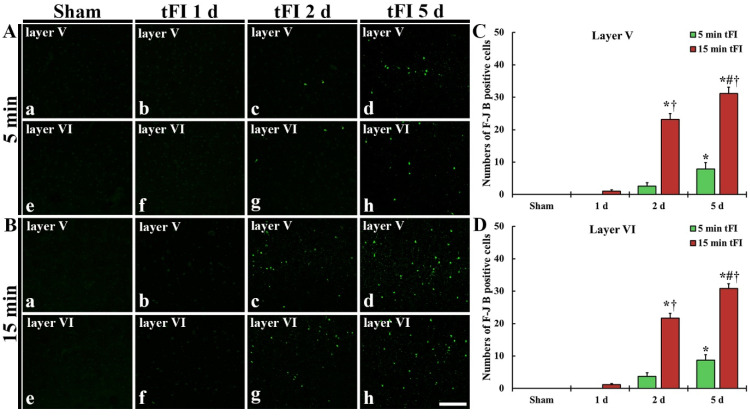
(**A**,**B**) F-J B histofluorescence staining in layers V and VI of the motor cortex in the mild sham (**Aa**,**Ae**), mild tFI (**Ab**–**Ad**,**Af**–**Ah**), severe sham (**Ba**,**Be**), and severe tFI (**Bb**–**Bd**,**Bf**–**Bh**) groups at one day (**Ab**,**Af**,**Bb**,**Bf**), two days (**Ac**,**Ag**,**Bc**,**Bg**) and five days (**Ad**,**Ah**,**Bd**,**Bh**) after tFI. A few F-J B-cells are detected in the mild tFI group two and five days after tFI. In contrast, many F-J B-cells are found in the severe tFI group two and five days after tFI. Scale bars = 200 μm. (**C**,**D**) Numbers of F-J B-cells in layers V and VI, respectively (*n* = 7, respectively; * *p* < 0.05, significantly different from mild and severe sham group, # *p* < 0.05, significantly different from pre-time point group and † *p* < 0.05, significantly different from mild tFI group at corresponding time point). The bars indicate the means ± SEM.

To examine tFI-induced cell damage in the motor cortex, CV staining was performed. CV-cells were similarly distributed in all layers (layer I-VI) in the motor cortex of all sham groups (Figure 2A,E). In the mild tFI group, CV-cells were apparently decreased in numbers and damaged in morphology in layers V and VI on day 5 after tFI (Figure 2D). In the severe tFI group, a slight decrease in CV-cell numbers and their morphological damage were shown in layers V and VI on day 2 after tFI (Figure 3G), and, on day 5 after tFI, a heavy decrease in CV-cell numbers and their damage were found in the layers (Figure 2H).

#### 2.2.2. Fluoro-Jade B (F-J B)-Positive Cells (F-J B-Cells) 

Histofluorescence staining using F-J B was performed to examine tFI-induced neuronal degeneration (death or loss) in the motor cortex. No F-J B-cells in the motor cortex were found in all sham groups (Figure 3Aa,Ae,Ba,Be). On day 1 after tFI, F-J B-cells in the mild tFI group were not shown at one day after tFI (Figure 3Ab,Af,C,D); however, a few F-J B-cells were detected in layers V and VI in the severe tFI group (Figure 3Bb,Bf,C,D). On day 2 after tFI, some F-J B-cells were found in the two layers in the mild tFI group (Figure 3Ac,Ag,C,D), and, in the severe tFI group, many F-B-cell were shown in the two layers (Figure 3Bc,Bg,C,D). On day 5 after tFI, the numbers of F-J B-cells was more increased in the two layers, showing that the F-J B-cell number of the severe tFI group was 3.9 and 3.5-fold, respectively, of the mild tFI group (Figure 3Ad,Ah,Bd,Bh,C,D).

### 2.3. Astrogliosis

#### 2.3.1. Glial Fibrillary Acidic Protein (GFAP)-Immunoreactive Astrocytes

**Figure 4:**
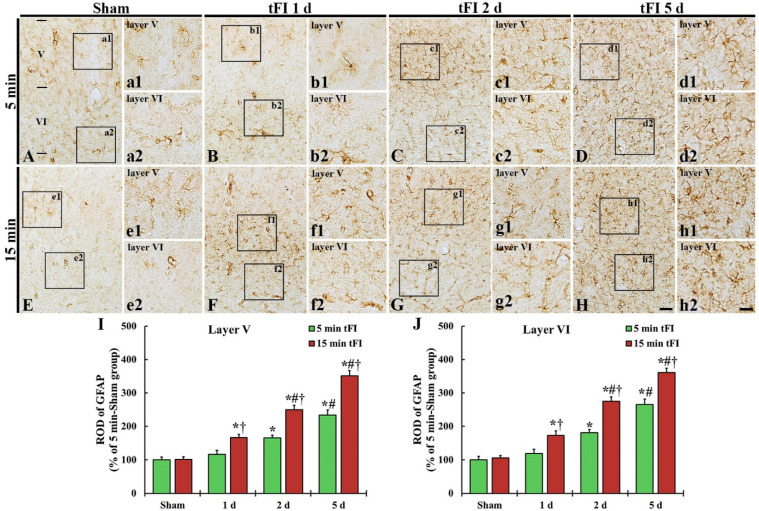
(**A**–**H**) GFAP immunohistochemistry in layers V and VI of the motor cortex in the mild sham (**A**,**a1**,**a2**), mild tFI (**B**–**D**,**b1**–**d1**,**b2**–**d2**), severe sham (**E**,**e1**,**e2**), and severe tFI (**F**–**H**,**f1**–**h2**,**f2**–**h2**) groups on day 1 (**B**,**b1**,**b2**,**F**,**f1**,**f2**), day 2 (**C**,**c1**,**c2**,**G**,**g1**,**g2**) and day 5 (**D**,**d1**,**d2**,**H**,**h1**,**h2**) after tFI. In the mild tFI group, GFAP immunoreactivity is increased from 2 days after tFI. However, GFAP immunoreactivity in the severe group is significantly enhanced from one day after tFI. On day 5 after tFI, the cell bodies and processes of GFAP-astrocytes are larger and thicker than those of the sham groups, showing that GFAP immunoreactivity in the severe tFI group is higher than that in the mild tFI group. Scale bars = 200 μm (**A**–**H**) and 50 μm (**a1**–**h2**). (**I**,**J**): ROD as % of GFAP-immunoreactive structures in layers V and VI (*n* = 5 or 7, respectively; * *p* < 0.05, significantly different from mild or severe sham group, # *p* < 0.05, significantly different from corresponding former time group, and † *p* < 0.05, significantly different from mild tFI group at the corresponding time). The bars indicate the means ± SEM.

GFAP immunohistochemistry was done to evaluate astrogliosis after tFI (Figure 4). GFAP-immunoreactive astrocytes (GFAP-astrocytes) with small cell bodies and thin processes were distributed throughout layers V and VI in the motor cortex of the mild and severe sham groups, showing that GFAP immunoreactivity was not different between the two sham groups (Figure 4A,E,I,J). GFAP immunoreactivity in the mild tFI group GFAP-astrocytes was slightly increased in the two layers on day 1 after tFI, but GFAP immunoreactivity in the severe group was significantly increased (167% for the sham group) at that point in time after tFI (Figure 4B,F,I,J). On day 2 after tFI, GFAP immunoreactivity in both groups was increased in the two layers (165% versus 250% in layer V and 180% versus 275% in layer VI) compared with the sham group (Figure 4C,G,I,J). On day 5 after tFI, GFAP immunoreactivity was increased (234% versus 351% in layer V and 265% versus 361% in layer VI compared with the sham group), showing that the GFAP immunoreactivity in the severe group was significantly higher (1.5-fold in layer V and 1.4-fold in layer VI) than that in the mild group (Figure 4D,H,I,J). At this point in time, the cell bodies and processes of GFAP-astrocytes became larger and thicker in both groups; however, the ends of their processes became blunt, which seemed to be damaged (collapsed) (Figure 4d1,d2,h1,h2). 

#### 2.3.2. GFAP Protein Level

**Figure 5:**
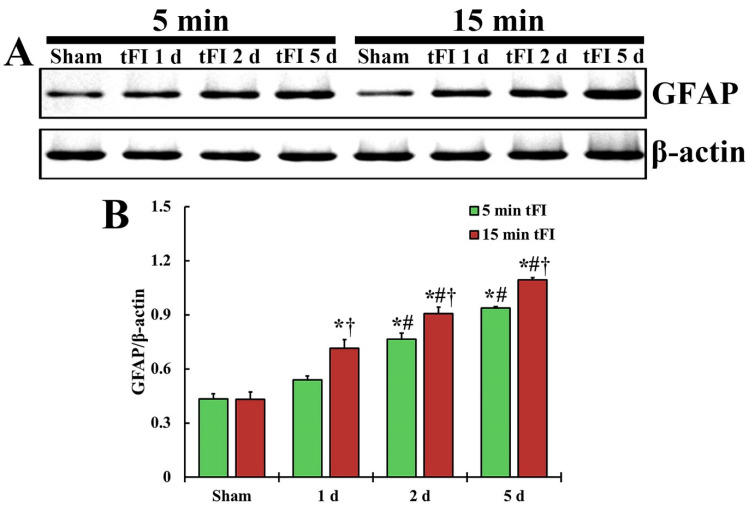
(**A**) Representative blot image and quantitative analysis of GFAP protein level in the motor cortex of the mild sham, mild tFI, severe sham and severe tFI groups on days 1, 2 and 5 after tFI. (**B**) Densitometric analysis of GFAP protein level by normalization to the level of β-actin. The bars indicate the means ± SEM (*n* = 5, respectively; * *p* < 0.05, significantly different from mild or severe sham group, # *p* < 0.05, significantly different from corresponding former time group, and † *p* < 0.05, significantly different from mild tFI group at the corresponding time). The bars indicate the means ± SEM.

The changing pattern of GFAP protein level in the motor cortex of both tFI groups was similar to the change in GFAP immunoreactivity (Figure 5). The GFAP level in the mild tFI group was significantly increased from two days after tFI and was 177% at two days and 216% at five after tFI compared with the sham group. The GFAP level in the severe tFI group was significantly increased from one day after tFI, showing that the level was 166% at one day, 210% at two days, and 253% at five days after tFI as compared to the mild tFI. 

#### 2.3.3. S100-Immunoreactive Astrocytes

**Figure 6:**
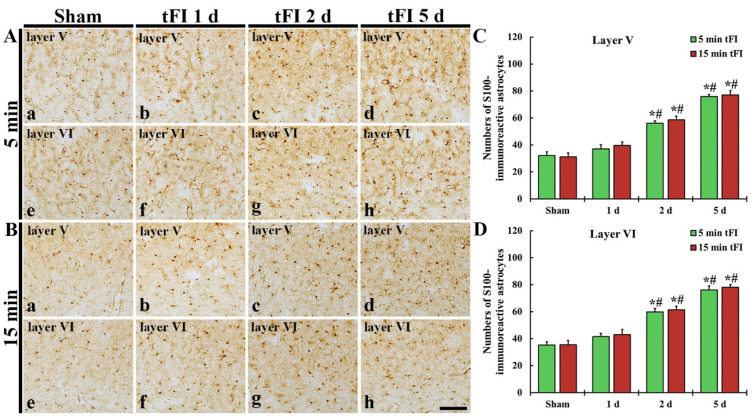
(**A**,**B**) S100 immunohistochemistry in layers V and VI of the motor cortex in the mild sham (**Aa**,**Ae**), mild tFI (**Ab**–**Ad**,**Af**–**Ah**), severe sham (**Ba**,**Be**), and severe tFI (**Bb**–**Bd**,**Bf**–**Bh**) groups at one day (**Ab**,**Af**,**Bb**,**Bf**), two days (**Ac**,**Ag**,**Bc**,**Bg**) and five days (**Ad**,**Ah**,**Bd**,**Bh**) after tFI. S100-astrocytes are significantly increased in numbers from two days after tFI. Scale bar = 200 μm. (**C,D**) Mean numbers of S100-astrocytes in layers V and VI (*n* = 5 or 7, respectively; * *p* < 0.05, significantly different from mild or severe sham group, # *p* < 0.05, and significantly different from corresponding former time group). The bars indicate the means ± SEM.

S100 immunohistochemistry was performed to evaluate the change in numbers of astrocytes after tFI (Figure 6). In both sham groups, S100-immunoreactive astrocytes (S100-astrocytes) were similarly detected in layers V and VI of the motor cortex (Figure 6Aa,Ae,Ba,Be,C,D). On day 1 after tFI, the numbers of S100-astrocytes were slightly (not significantly) increased in both tFI groups (Figure 6Ab,Af,Bb,Bf,C,D). However, the numbers of S100-astrocytes in both layers were gradually and significantly increased in both groups from two days after tFI, showing that no significant difference in the numbers was shown between the two groups (Figure 6Ac,Ad,Ag,Ah,Bc,Bd,Bg,Bh,C,D): on day 5 after tFI, the number of the astrocytes was 2.4-fold in layer V and 2.2-fold in layer VI as compared to the sham group (Figure 6C,D). 

### 2.4. Damage to Astrocyte Endfoot

#### 2.4.1. Double Immunofluorescence for GFAP and Glucose Transporter 1 (GLUT-1)

**Figure 7:**
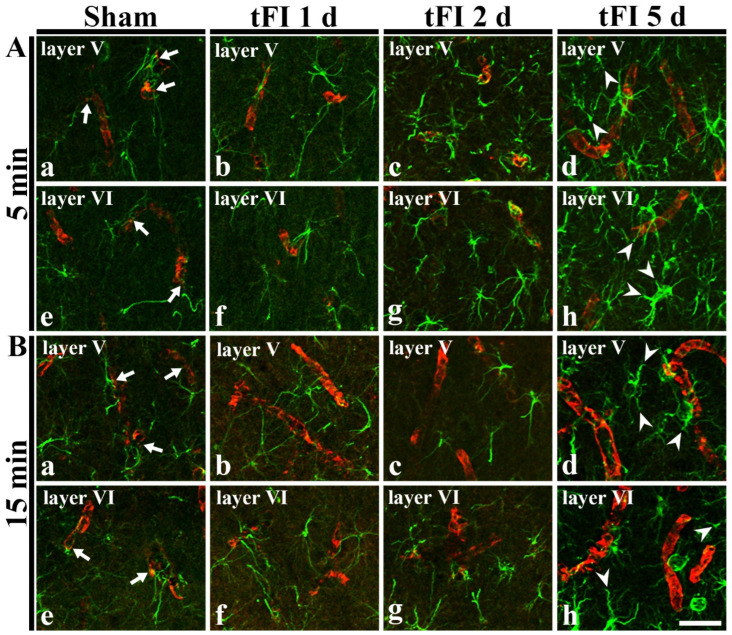
Double immunohistofluorescence for AEf around blood vessels in layers V and VI using anti-GFAP (green) and GLUT-1 (red) in the mild (**A**) sham (**Aa**,**Ae**), mild tFI (**Ab**–**Ad**,**Af**–**Ah**), severe (**B**) sham (**Ba**,**Be**), and severe tFI (**Bb**–**Bd**,**Bf**–**Bh**) groups at one day (**Ab**,**Af**,**Bb**,**Bf**), two days (**Ac**,**Ag**,**Bc**,**Bg**) and five days (**Ad**,**Ah**,**Bd**,**Bh**) after tFI. In all sham groups, GFAP-AEf (arrows) contact GLUT-1-endothelial cells. On day 5 after tFI, GFAP-AEf (arrowheads) around GLUT-1-endothelial cells apparently disappear in both groups. Scale bars = 50 μm.

In GFAP immunohistochemical findings at five days after tFI, the ends of GFAP-astrocyte processes became blunt. To examine the tFI-induced changes of astrocyte endfeet (AEf), we performed double immunofluorescence for GFAP and GLUT-1. In both sham groups, GFAP-immunoreactive AEf (GFAP-AEf) were detected around GLUT-1-immunoreactive endothelial cells (Figure 7Aa,Ae,Ba,Be). On day 1 after tFI, GFAP-AEf were not significantly altered in both groups (Figure 7Ab,Af,Bb,Bf). On day 2 after tFI, GFAP-AEf tended to be blunt (damaged) altered in the severe tFI groups (Figure 7Ac,Ag,Bc,Bg). On day 5 after tFI, GFAP-AEf were apparently lost (damaged) around GLUT-1-endothelial cells in both tFI groups (Figure 7Ad,Ah,Bd,Bh).

#### 2.4.2. Ultrastructural Finding

**Figure 8:**
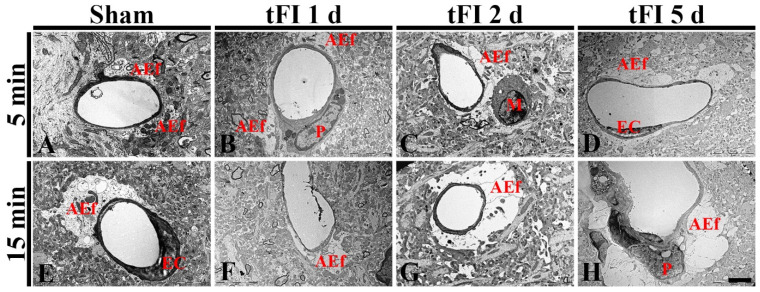
Electron micrographs of AEf around a blood vessel using TEM in the mild sham (**A**), mild tFI (**B**–**D**), severe sham (**B**), and severe tFI (**F**–**H**) groups at one day (**B**,**F**), two days (**C**,**G**) and five days (**D**,**H**) after tFI. In both sham groups, AEf has many mitochondria and surrounds the basal membrane of endothelial cells (EC). AEf becomes damaged with time after tFI: in the severe tFI group, the damage is more significant. On day 5 after tFI, AEf looks like a vacuole and no mitochondria were in both groups: in the severe group, EC and pericyte (P) are severely damaged. Scale bar = 2.5 μm.

To confirm the tFI-induced damage of AEf, we investigated the changes of AEf in layers V/VI after tFI using a transmission electron microscope (TEM) (Figure 8). In both sham groups, the fine structure of AEf was easily distinguished: AEf had many mitochondria and surrounded the basal membrane of endothelial cells, which were relatively thin (Figure 8A,E). However, the structure of the AEf was markedly changed with time after tFI. On day 1 after tFI, AEf became pale in the mild tFI group (Figure 8B); in contrast, AEf in the severe tFI group was paler and had fewer mitochondria than in the mild tFI group (Figure 8F). On day 2 after tFI, AEf in the mild tFI group became paler and had fewer mitochondria, and, fortunately, we found that microglia (as resident macrophage cells) contacted AEf (Figure 8C). In the severe tFI group, AEf was almost destroyed, and mitochondria were severely damaged (Figure 8G). On day 5 after tFI, AEf looked like a vacuole and with no mitochondria in both groups, showing that endothelial cells and pericytes were severely damaged in the severe tFI group (Figure 8D,H).

## 3. Discussion

Histopathological assessment has revealed neuronal loss (death) in vulnerable regions in the forebrain, such as the striatum, sensory and motor cortex, and hippocampus after tFI [6,7,8,13]. Further, tFI in gerbils can lead to motor hyperactivity, such as an increase in locomotion and rotation. The most remarkable change in motor behaviors is shown one day after tFI in gerbils [3,26]. In the present study, one day after tFI, SMA was significantly increased in the mild tFI group compared to that in the sham group. In addition, SMA in the severe tFI group was significantly higher (1.6-fold) than that in the mild tFI group. These findings indicate that increased SMA is closely associated with a transient response following tFI.

The degree of neuronal damage or death (loss) in the forebrain following IR injury is different according to the brain structure or regions. Studies using monkeys [27] and rats [28,29] have shown that neurons in the cerebral motor cortex are damaged following IR injury in the forebrain. However, these neurons are significantly more resistant to IR injury than neurons located in the hippocampal CA1 region. In addition, neuronal loss (death) in the motor cortex is more delayed than that in the mouse hippocampus after IR injury [30]. Moreover, cerebral cortical injury in the forebrain following IR is significantly different according to the duration of tFI. For example, in a mouse model of transient global cerebral ischemia, Chen et al. (2009) have shown that the severity of cortical injury tends to be increased according to ischemic duration [12]. Additionally, Murakami et al. (1998) have reported that tissue injury in the cerebral cortex is more severe after increasing ischemic duration in a rat model of transient focal cerebral ischemia [31]. In our current study, tFI-induced neuronal death occurred mainly in layers V and VI of the motor cortices of both mild (5-min) and severe (15-min) tFI groups, with neuronal loss in the severe tFI group being significantly higher than that in the mild tFI group. Studies using gerbils have shown that the number of neurons located in the cerebral motor cortex is significantly reduced compared to that in the sham group after tFI is induced by unilateral or bilateral common carotid artery occlusion [5,25,26]. In particular, Ahn et al. (2019) reported that many and numerous F-J B-cells (dead cells) were detected in layers II-III and V-VI, respectively, in the gerbil motor cortex on day 5 after 30 min of ligation of the unilateral common carotid artery [4]. Furthermore, in gerbil models of tFI for 5 min and 15 min, the number of F-J B-cells in the somatosensory cortex, following a 15-min tFI, is significantly higher than that in the 5-min tFI [7]. Findings of the present study indicate that tFI-induced neuronal death in the motor cortex occurs layer-specifically and primarily in the deep layers and that tFI-induced neuronal death in the deep layers might occur even earlier and more severely in the severe tFI group than in the mild tFI group. 

Astrogliosis is also referred to as reactive astrogliosis, which is an abnormal increase in the number of astrocytes with the destruction of neurons due to CNS damages or diseases [32,33]. Other terms, such as “activation of astrocytes” or “activated astrocytes” are used to refer to astrocyte responses to damage or diseases in the CNS [33]. In this paper, we used “astrogliosis” and “reactive astrocytes” for astrocyte responses associated with tFI. The position of reactive astrocytes has been a controversial subject. According to many studies, reactive astrocytes have double-sidedness (both beneficial and harmful effects) [32,34]. Emerging data in ischemic stroke suggest that reactive astrocytes can exert both beneficial and detrimental effects following an ischemic stroke: (1) reactive astrocytes can provide neuroprotective effects and contribute to the restoration, and (2) they can also secrete inflammatory modulators, leading to exacerbation of an ischemic lesion [3]. Some studies have shown that, in gerbil models of IR injury, GFAP-astrocytes are hypertrophied and significantly increased in their number in the ipsilateral primary motor cortex on day 5 after unilateral common carotid artery occlusion [4]. In addition, reactive astrocytes are more significantly increased in the hippocampus after a longer duration of tFI [4,13]. Lee et al. (2013) have reported that GFAP expression in astrocytes is stronger in the gerbil somatosensory cortex after a 15-min tFI than that after a 10-min tFI, indicating that astrogliosis and neuronal death/damage were apparently increased following a longer time of IR [7]. In the present study, GFAP expression in the gerbil motor cortex was significantly higher in severe tFI than in mild tFI. However, only a few papers have shown that the degeneration of astrocytes occurs in ischemic areas following IR injury. Ito et al. (2009) reported that the processes of astrocytes were degenerated in cerebral cortical regions after IR injury in gerbils and suggested that the heterogeneity of their disintegration might be closely associated with selective neuronal loss (death) following IR injury [35]. In the present study, tFI-induced changes of astrocytes in layers V and VI, where neuronal death occurred in both tFI groups, showed that GFAP-astrocytes became enlarged and GFAP immunoreactivity was significantly higher in the severe tFI group than in the mild tFI group; however, numbers of astrocyte processes were significantly reduced and the ends of these processes seemed to be cut (blunt). In addition, the number of S100-astrocytes was gradually increased in layers V and VI after tFI. This finding could be supported by previous studies reporting that the proliferation of astrocytes occurs within a few days after ischemic insults and that proliferating astrocytes play a critical role in the development of astroglial scar [36,37,38]. 

It is well known that the BBB in normal CNS is composed of four main cellular elements: endothelial cells, AEf, pericytes and microglial cells. In particular, AEf can support capillary endothelial cells to contribute to BBB integrity in normal CNS. Disruption of BBB components and/or BBB damage can occur following an ischemic injury [39,40]. Therefore, maintenance of BBB integrity has been thought to be very important to reduce brain damage following an ischemic injury [41,42,43]. As described above, in this study, ends of GFAP-astrocyte processes (AEf) were blunt at five days after tFI. When they (AEf) were examined around capillaries using double immunofluorescence for GFAP/GLUT-1 and TEM, they were damaged early and severely in the mild tFI group after tFI when compared with the severe tFI group. Mitochondria in AEf were more severely destroyed in the severe tFI group than in the mild tFI group. AEf in both groups was hardly detected at five days after tFI and from two days after tFI, respectively. For the change of mitochondria following ischemic insults, it has been reported that mitochondria in the astrocyte processes degenerate in cerebral cortical regions after IR injuries in gerbils [35].

## 4. Materials and Methods

### 4.1. Animals

The protocol of all animal experiments in this study was approved (approval no., KW-2000113-1) on 13 January 2020 by the Ethics Committee of Kangwon National University (Chuncheon, Korea). We did our best to minimize animal suffering during the whole experiment. We used male gerbils (*Meriones unguiculatus*; six months of age, 75–85 g of body weight) in order to minimize the effects of hormones. They were housed in a pathogen-free environment under standard conditions, such as controlled temperature (about 23 °C) and humidity (about 60%) on a 12:12 h light–dark cycle.

**Figure 9:**
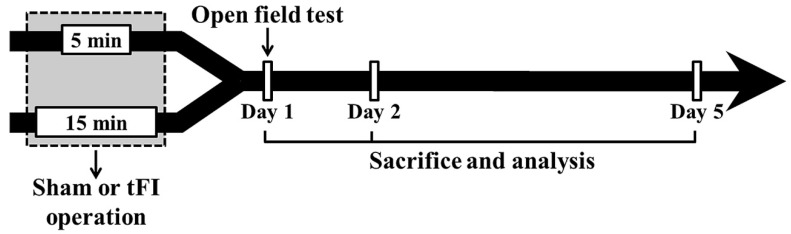
Experimental procedure. Gerbils are given five- and 15-min tFI, respectively, for mild and severe ischemia and reperfusion injury. Open field test (for change of motor activity) was performed on day 1 after tFI, and the gerbils were sacrificed on days 1, 2 and 5 after tFI for the examination of histopathological and biochemical changes.

The gerbils (total *n* = 142) were grouped as follows: (1) mild sham group (*n* = 26) which was given the sham tFI operation for five minutes, (2) mild tFI group (*n* = 45) which was given the tFI operation for five minutes, (3) severe sham group (*n* = 26) which was given the sham tFI operation for 15 min, and (4) severe tFI group (*n* = 45) which was given the tFI operation for 15 min. In all tFI groups, the gerbils (*n* = 7, respectively, for immunohistochemistry; *n* = 5, respectively, for Western blotting; *n* = 3, respectively, for ultrastructure) were sacrificed on one day, two days and five days after tFI (Figure 9). In all sham groups, the gerbils (*n* = 5, respectively, for immunohistochemistry; *n* = 5, respectively, for Western blotting; *n* = 3, respectively, for ultrastructure) were sacrificed at 0 h and five days after sham tFI to reduce the numbers.

### 4.2. Induction of tFI

The surgical procedure of tFI was carried out, as described in our previous study [13]. In short, the gerbils were anesthetized with isoflurane (4 L/min, 2.5%). Under the anesthesia, bilateral common carotid arteries (BCCA) were isolated from the carotid sheath and ligated with clips (0.69 N; Yasargil FE 723 K; Aesculap Inc., Tuttlingen, Germany) for five minutes to develop mild tFI and 15 min to develop severe tFI. Using a HEINE K180 ophthalmoscope (Heine Optotechnik, Herrsching, Germany), right and left central arteries located in the retinae were observed in order to confirm the complete blockade of BCCA. The clips were removed after perfect tFI. During the tFI induction, body temperature was controlled at normothermic conditions (37 ± 0.1 °C) using a TR-100 rectal temperature probe (Fine Science Tools, Foster City, CA, USA). For the sham tFI operation, an identical surgical procedure was carried out without BCCA ligation. After the sham and tFI operation, the operated gerbils were kept under 24 ± 1 °C temperature and 55 ± 5% relative humidity.

### 4.3. Open Field Test

The open field test is a test used to evaluate general locomotor activity levels in animals (usually rodents) in scientific research. In this experiment, SMA was evaluated to investigate the change in tFI-induced hyperactivity one day after tFI. As described previously [13], each gerbil was placed in the open field cage (44 cm at its width, 44 cm at its length, and 30 cm at its height; Ugo Basile S.R.L., Gemonio, Italy) for one hour. The total traveled distance was recorded using an Ethovision XT 9 automatic video system (Noldus Information Technology, Wageningen, The Netherlands). In this experiment, the cage was wiped with 70% ethyl alcohol after each test to obliterate body odor, which could affect the movement of the other gerbils.

### 4.4. Tissue Preparation for Histopathology

Brain tissue sections were prepared, as previously described [4,44]. In brief, the gerbils were deeply anesthetized by intraperitoneal injection of 200 mg/kg pentobarbital sodium. Under the deep anesthesia, the gerbils were washed (six mL/min of flow rate and 60 mL of total perfused volume) with 0.1 M phosphate-buffered saline (pH 7.4) and fixed with 4% paraformaldehyde (in 0.1 M phosphate buffer, pH 7.4). The removed brains were more fixed in the same fixative and infiltrated with 25% sucrose to protect the tissue from freezing-induced damage. Using an SM2010 R sliding microtome (Leica Biosystems, Wetzlar, Germany) equipped with a BFS-40 MP freezing stage (Physitemp Instruments Inc., Clifton, NJ, USA), 30-μm coronal sections were made.

### 4.5. Stainings for Cell Damage/Death

#### 4.5.1. CV Histochemistry

CV staining was conducted to investigate cellular change (damage) in the motor cortex following tFI. As previously described [4], the sections were incubated in 0.1% CV acetate (Sigma-Aldrich Co., St. Louis, MO, USA) for 25 min at room temperature. After washing, the sections were decolorized in 50% ethyl alcohol for five minutes. Thereafter, the sections were dehydrated through a series of ethanol (ending in 100% ethanol) and cleared in xylene. Lastly, the stained sections were coverslipped with Canada balsam (Kanto Chemical Co., Inc., Tokyo, Japan).

The CV-stained cells were captured using a BX53 microscope (Olympus, Tokyo, Japan) equipped with a DP72 digital camera (Olympus, Tokyo, Japan) and cellSens imaging software (Olympus, Tokyo, Japan).

#### 4.5.2. F-J B Histofluorescence

F-J B histofluorescence was carried out to examine tFI-induced neuronal loss (death) in the motor cortex. As described in previous studies [4,45], in brief, the sections were drenched in 0.06% potassium permanganate (Sigma-Aldrich Co., St. Louis, MO, USA) for 30 min at 25 °C and concisely rinsed with distilled water. Thereafter, the sections were incubated with 0.0004% F-J B (Histo-chem Inc., Jefferson, AR, USA) in 0.1% acetic acid for 40 min at room temperature. After washing, the sections were fully dried in a WiseVen^®^ WOC High Clean Air Oven (Daihan Scientific Co Ltd., Wonju, Korea) at 45 °C for six hours. Finally, the sections were cleared in xylene and coverslipped with dibutyl phthalate polystyrene xylene (DPX; Sigma-Aldrich Co., St. Louis, MO, USA).

To count the F-J B positive cells, five sections per gerbils were used. According to a previous report [46], the F-J B positive cells were observed using a BX53 epifluorescent microscope (Olympus, Tokyo, Japan). The F-J B positive cells were captured and counted in 140 × 140 μm at the center of layers V and VI, respectively. The mean number of F-J B cells was calculated using NIH Image 1.59 software (NIH, Bethesda, Rockville, MD, USA).

#### 4.5.3. Immunohistochemistry for Astrocytes

Immunohistochemical staining was performed to investigate changes in astrocytes in the motor cortex following tFI. In brief, as described using the avidin-biotin complex method [4,45], the sections were washed with 0.1 M phosphate-buffered saline (pH 7.4) and immersed in 0.3% hydrogen peroxide for 20 min at room temperature to reduce endogenous peroxidase activity. After washing, the sections were incubated in 5% normal horse or goat serum for 30 min at room temperature in order to block non-specific immunoreaction. After washing, the sections were immunoreacted with primary antibodies (GFAP and S100 protein) for astrocytes (Table 1) for 48 h at 4℃. Subsequently, the sections were incubated in each biotinylated secondary antibody (Table 1) for two hours at room temperature followed by avidin-biotin complex (diluted 1:300; Vector Laboratories, Burlingame, CA, USA) for two hours at room temperature. After washing, the sections were soaked in 0.06% 3,3′-diaminobenzidine tetrahydrochloride (DAB; Sigma-Aldrich Co, St Louis, MO, USA) (in 100 mM phosphate-buffered saline containing 0.1% H_2_O_2_). Finally, the sections were dehydrated through a series of ethanol and cleared in xylene, and coverslipped with Canada balsam (Kanto Chemical Co., Inc., Tokyo, Japan).

To quantitatively evaluate the immunoreactivity of GFAP-immunostained structure, its digital image was taken using a BX53 upright microscope (Olympus, Japan) and analyzed according to our published protocol [4]. Briefly, the captured GFAP-immunostained structure was represented as relative optical density (ROD) using Adobe Photoshop 8.0 (Adobe Inc., San Jose, CA, USA) and NIH Image software 1.59 (NIH, Bethesda, MD, USA). ROD was calibrated as % compared to the sham group (100%).

The numbers of S100-immunostained cells were evaluated, as previously described [21]. Briefly, the digital images of the cells were captured using a BX53 upright microscope (Olympus, Japan). The captured cells were counted and evaluated using Optimas 6.5 (an image analyzing system; CyberMetrics, Scottsdale, AZ, USA).

### 4.6. Western Blotting of GFAP

Western blot analysis for GFAP was performed in the motor cortex according to previously described methods [8,45]. In short, the gerbils were deeply anesthetized by intraperitoneal administration of 200 mg/kg pentobarbital sodium and sacrificed. Immediately, their brains were removed, and cortical tissues were collected, homogenized and total protein was extracted using RIPA buffer (CoWin Biosciences Inc., Cambridge, MA, USA). The BCA Protein Assay kit (QuantiPro™, Sigma-Aldrich Inc., St Louis, MO, USA) was used in order to measure the GFAP protein concentration. Total protein (30 μg) was separated using 10% SDS-PAGE and then transferred onto nitrocellulose membranes of Pall Co. (East Hills, NY, USA). To block non-specific staining, the membranes were incubated in 5% defatted milk for 60 min. Subsequently, the membranes were immunoreacted with mouse anti-GFAP (diluted 1:1100; Chemicon International, Temecula, CA, USA) for 24 h at 4 °C and then incubated in peroxidase-conjugated secondary antibody as donkey anti-mouse IgG (diluted 1:4000; Santa Cruz Biotechnology, Santa Cruz, CA, USA) for 60 min. The signals were developed using a luminol-based chemiluminescence kit by Pierce (Thermo Fisher Scientific Inc., Waltham, MA, USA). 

The protein bands were visualized using a Bio-Rad imaging system obtained from Bio-Rad Laboratories Inc. (Hercules, CA, USA) and quantified using ImageJ (version 1.52) of the National Institutes of Health (Bethesda, MD, USA).

### 4.7. Double Immunofluorescence for GFAP/GLUT-1

To distinguish astrocyte endfeet from endothelial cells in BBB, double immunofluorescence was conducted, as previously described [8,47]. In brief, the sections were immunostained with primary antibodies: mouse anti-GFAP (diluted 1:1000; Merck-Millipore, Burlington, MA, USA) and rabbit anti-GLUT-1 (a marker for endothelial cells) (diluted 1:100; Chemicon, Temecula, CA, USA) at 4 °C for nine hours. After briefly washing, the sections were incubated in the mixture of goat anti-rabbit IgG conjugated with IgG Alexa Fluor^®^ 546 (diluted 1:500; Invitrogen, Waltham, MA, USA) and donkey anti-mouse IgG conjugated with Alexa Fluor^®^ 488 (diluted 1:500; Invitrogen). Thereafter, the sections were washed and dehydrated. The immunostained sections were finally coverslipped with DPX (Sigma-Aldrich Co., St. Louis, MO, USA).

The double immunoreaction of GFAP and GLUT-1 was observed using a Zeiss LSM510 confocal microscope (Carl Zeiss, Oberkochen, Germany), which was located in the Korea Basic Science Institute (KBSI; Chuncheon, Korea).

### 4.8. Ultrastructural Examination of Astrocyte Endfeet

The gerbils were perfused intracardially with normal saline and fixed rapidly with 4% glutaraldehyde (in phosphate-buffered saline) at 4 °C. The motor cortices were isolated and post-fixed with 1% osmium tetroxide for two hours. Subsequently, the tissues were dehydrated through the ethanol series and acetone and embedded in Epon812 epoxy resin (PELCO Eponate 12TM Kit; Clovis, CA, USA) according to general methods. Thereafter, the embedded tissues were cut into 50-nm thickness using a UC-7 ultramicrotome (Leica Biosystems, Wetzlar, Germany) and placed on copper grids. Thereafter, the sections were stained using uranyl acetate, and ultrastructural changes following tFI were observed using a Philips EM400 transmission electron microscope (Koninklijke Philips N.V., Amsterdam, The Netherlands).

### 4.9. Statistical Analysis

Statistical analyses were performed using SPSS software 15.0 (SPSS Inc., Chicago, IL, USA) for statistical analysis. In addition, the Kolmogorov–Smirnov test was used to measure normal distributions, and Bartlett’s test was used to evaluate the identical standard error of the mean (SEM). Furthermore, all data were taken for the normality test. The statistical significances of the means were determined by two-way ANOVA, followed by a post hoc Tukey’s test for all pairwise multiple comparisons. A value of *p*-value less than 0.05 was considered significant.

## 5. Conclusions

In conclusion, this study showed that astrogliosis (reactive astrocytes) occurred before neuronal damage/death in layers V and VI of gerbil cerebral motor cortices after tFI and that tFI-induced changes were more advanced in severe tFI than in mild tFI. In particular, AEf damage/disruption preceded neuronal damage/death in both mild and severe tFI, showing that AEf damage following severe tFI was more significant than that following mild tFI. These results indicate that the severity of neuronal damage/loss following ischemic insults might be closely associated with the degree of AEf damage/disruption under various ischemic impacts.

## Figures and Tables

**Table 1:** Primary and secondary antibodies for immunohistochemical staining.

Primary Antibodies	Dilution	Suppliers
Mouse anti-glial fibrillary acidic protein (GFAP)	1:800	Merck-Millipore, Burlington, MA, USA
Rabbit anti-S100 protein (S100)	1:1000	Abcam, Cambridge, UK
**Secondary Antibodies**	**Dilution**	**Suppliers**
Biotinylated horse anti-mouse IgG	1:250	Vector Laboratories Inc., Burlingame, CA, USA
Biotinylated goat anti-rabbit IgG	1:250	Vector Laboratories Inc., Burlingame, CA, USA

## Data Availability

The data presented in this study are available on request from the corresponding author.

## Abbreviations

AEf	astrocyte endfoot
BBB	blood–brain barrier
CNS	central nervous system
F-J B	Fluoro-Jade B
GFAP	glial fibrillary acidic protein
GLUT-1	glucose transporter 1
IR	ischemia and reperfusion
ROD	relative optical density
SMA	spontaneous motor activity
tFI	transient forebrain ischemia
TEM	transmission electron microscope

## References

[1] Kondo T., Yoshida S., Nagai H., Takeshita A., Mino M., Morioka H., Nakajima T., Kusakabe K.T., Okada T. (2018). Transient forebrain ischemia induces impairment in cognitive performance prior to extensive neuronal cell death in mongolian gerbil (meriones unguiculatus). J. Vet. Sci..

[2] Lipton P. (1999). Ischemic cell death in brain neurons. Physiol. Rev..

[3] Radenovic L., Selakovic V., Olivan S., Calvo A.C., Rando A., Janac B., Osta R. (2014). Neuroprotective efficiency of tetanus toxin c fragment in model of global cerebral ischemia in mongolian gerbils. Brain Res. Bull..

[4] Ahn J.H., Song M., Kim H., Lee T.K., Park C.W., Park Y.E., Lee J.C., Cho J.H., Kim Y.M., Hwang I.K. (2019). Differential regional infarction, neuronal loss and gliosis in the gerbil cerebral hemisphere following 30 min of unilateral common carotid artery occlusion. Metab. Brain Dis..

[5] de Araujo F.L., Bertolino G., Goncalves R.B., Marini Lde C., Coimbra N.C., de Araujo J.E. (2012). Neuropathology and behavioral impairments after three types of global ischemia surgery in meriones unguiculatus: Evidence in motor cortex, hippocampal ca1 region and the neostriatum. J. Neurol. Sci..

[6] Kirino T., Sano K. (1984). Selective vulnerability in the gerbil hippocampus following transient ischemia. Acta Neuropathol..

[7] Lee J.C., Ahn J.H., Lee D.H., Yan B.C., Park J.H., Kim I.H., Cho G.S., Kim Y.M., Lee B., Park C.W. (2013). Neuronal damage and gliosis in the somatosensory cortex induced by various durations of transient cerebral ischemia in gerbils. Brain Res..

[8] Lee T.K., Lee J.C., Kim J.D., Kim D.W., Ahn J.H., Park J.H., Kim H.I., Cho J.H., Choi S.Y., Won M.H. (2021). Populus tomentiglandulosa extract is rich in polyphenols and protects neurons, astrocytes, and the blood-brain barrier in gerbil striatum following ischemia-reperfusion injury. Molecules.

[9] Kim M.J., Cho J.H., Cho J.H., Park J.H., Ahn J.H., Tae H.J., Cho G.S., Yan B.C., Hwang I.K., Lee C.H. (2015). Impact of hyperthermia before and during ischemia-reperfusion on neuronal damage and gliosis in the gerbil hippocampus induced by transient cerebral ischemia. J. Neurol. Sci..

[10] Liu F., McCullough L.D. (2012). Interactions between age, sex, and hormones in experimental ischemic stroke. Neurochem. Int..

[11] Manwani B., Liu F., Scranton V., Hammond M.D., Sansing L.H., McCullough L.D. (2013). Differential effects of aging and sex on stroke induced inflammation across the lifespan. Exp. Neurol..

[12] Chen B., Friedman B., Cheng Q., Tsai P., Schim E., Kleinfeld D., Lyden P.D. (2009). Severe blood-brain barrier disruption and surrounding tissue injury. Stroke.

[13] Lee T.K., Kim H., Song M., Lee J.C., Park J.H., Ahn J.H., Yang G.E., Kim H., Ohk T.G., Shin M.C. (2019). Time-course pattern of neuronal loss and gliosis in gerbil hippocampi following mild, severe, or lethal transient global cerebral ischemia. Neural. Regen. Res..

[14] An H., Lee H., Yang S., Won W., Lee C.J., Nam M.H. (2021). Adenovirus-induced reactive astrogliosis exacerbates the pathology of parkinson’s disease. Exp. Neurobiol..

[15] Huang P.S., Tsai P.Y., Yang L.Y., Lecca D., Luo W., Kim D.S., Hoffer B.J., Chiang Y.H., Greig N.H., Wang J.Y. (2021). 3,6′-dithiopomalidomide ameliorates hippocampal neurodegeneration, microgliosis and astrogliosis and improves cognitive behaviors in rats with a moderate traumatic brain injury. Int. J. Mol. Sci..

[16] Pekny M., Pekna M. (2014). Astrocyte reactivity and reactive astrogliosis: Costs and benefits. Physiol. Rev..

[17] Sofroniew M.V. (2009). Molecular dissection of reactive astrogliosis and glial scar formation. Trends Neurosci..

[18] Ben Haim L., Rowitch D.H. (2017). Functional diversity of astrocytes in neural circuit regulation. Nat. Rev. Neurosci..

[19] Hamilton N.B., Attwell D. (2010). Do astrocytes really exocytose neurotransmitters?. Nat. Rev. Neurosci..

[20] Sofroniew M.V., Vinters H.V. (2010). Astrocytes: Biology and pathology. Acta Neuropathol..

[21] Kim H., Park J.H., Shin M.C., Cho J.H., Lee T.K., Kim H., Song M., Park C.W., Park Y.E., Lee J.C. (2019). Fate of astrocytes in the gerbil hippocampus after transient global cerebral ischemia. Int. J. Mol. Sci..

[22] Liu D., Smith C.L., Barone F.C., Ellison J.A., Lysko P.G., Li K., Simpson I.A. (1999). Astrocytic demise precedes delayed neuronal death in focal ischemic rat brain. Brain Res. Mol. Brain Res..

[23] Ouyang Y.B., Voloboueva L.A., Xu L.J., Giffard R.G. (2007). Selective dysfunction of hippocampal ca1 astrocytes contributes to delayed neuronal damage after transient forebrain ischemia. J. Neurosci..

[24] de Oliveira J.L., Crispin P., Duarte E.C., Marloch G.D., Gargioni R., Trentin A.G., Alvarez-Silva M. (2014). Histopathology of motor cortex in an experimental focal ischemic stroke in mouse model. J. Chem. Neuroanat..

[25] Kitabatake T.T., Marini L.C., Goncalves R.B., Bertolino G., de Souza H.C.D., de Araujo J.E. (2015). Behavioral effects and neural changes induced by continuous and not continuous treadmill training, post bilateral cerebral ischemia in gerbils. Behav. Brain Res..

[26] Bertolino G., De Araujo F.L., Souza H.C., Coimbra N.C., De Araujo J.E. (2013). Neuropathology and behavioral impairments after bilateral global ischemia surgery and exposure to static magnetic field: Evidence in the motor cortex, the hippocampal ca1 region and the neostriatum. Int. J. Radiat. Biol..

[27] Zhu H., Yoshimoto T., Imajo-Ohmi S., Dazortsava M., Mathivanan A., Yamashima T. (2012). Why are hippocampal ca1 neurons vulnerable but motor cortex neurons resistant to transient ischemia?. J. Neurochem..

[28] Guo H., Zhu L., Tang P., Chen D., Li Y., Li J., Bao C. (2021). Carthamin yellow improves cerebral ischemiareperfusion injury by attenuating inflammation and ferroptosis in rats. Int. J. Mol. Med..

[29] Kumar G., Mukherjee S., Paliwal P., Singh S.S., Birla H., Singh S.P., Krishnamurthy S., Patnaik R. (2019). Neuroprotective effect of chlorogenic acid in global cerebral ischemia-reperfusion rat model. Naunyn Schmiedebergs Arch. Pharmacol..

[30] Thal S.C., Thal S.E., Plesnila N. (2010). Characterization of a 3-vessel occlusion model for the induction of complete global cerebral ischemia in mice. J. Neurosci. Methods.

[31] Murakami K., Kondo T., Kawase M., Chan P.H. (1998). The development of a new mouse model of global ischemia: Focus on the relationships between ischemia duration, anesthesia, cerebral vasculature, and neuronal injury following global ischemia in mice. Brain Res..

[32] Shen X.Y., Gao Z.K., Han Y., Yuan M., Guo Y.S., Bi X. (2021). Activation and role of astrocytes in ischemic stroke. Front. Cell Neurosci..

[33] Sofroniew M.V. (2014). Astrogliosis. Cold Spring Harb. Perspect. Biol..

[34] Patabendige A., Singh A., Jenkins S., Sen J., Chen R. (2021). Astrocyte activation in neurovascular damage and repair following ischaemic stroke. Int. J. Mol. Sci..

[35] Ito U., Hakamata Y., Kawakami E., Oyanagi K. (2009). Degeneration of astrocytic processes and their mitochondria in cerebral cortical regions peripheral to the cortical infarction: Heterogeneity of their disintegration is closely associated with disseminated selective neuronal necrosis and maturation of injury. Stroke.

[36] Barreto G.E., Sun X., Xu L., Giffard R.G. (2011). Astrocyte proliferation following stroke in the mouse depends on distance from the infarct. PLoS ONE.

[37] Shimada I.S., Borders A., Aronshtam A., Spees J.L. (2011). Proliferating reactive astrocytes are regulated by notch-1 in the peri-infarct area after stroke. Stroke.

[38] Sims N.R., Yew W.P. (2017). Reactive astrogliosis in stroke: Contributions of astrocytes to recovery of neurological function. Neurochem. Int..

[39] Lin C.Y., Chang C., Cheung W.M., Lin M.H., Chen J.J., Hsu C.Y., Chen J.H., Lin T.N. (2008). Dynamic changes in vascular permeability, cerebral blood volume, vascular density, and size after transient focal cerebral ischemia in rats: Evaluation with contrast-enhanced magnetic resonance imaging. J. Cereb. Blood Flow Metab..

[40] Ma F., Sun P., Zhang X., Hamblin M.H., Yin K.J. (2020). Endothelium-targeted deletion of the mir-15a/16-1 cluster ameliorates blood-brain barrier dysfunction in ischemic stroke. Sci. Signal..

[41] Lee T.K., Kang I.J., Sim H., Lee J.C., Ahn J.H., Kim D.W., Park J.H., Lee C.H., Kim J.D., Won M.H. (2021). Therapeutic effects of decursin and angelica gigas nakai root extract in gerbil brain after transient ischemia via protecting bbb leakage and astrocyte endfeet damage. Molecules.

[42] Wang Z., Leng Y., Tsai L.K., Leeds P., Chuang D.M. (2011). Valproic acid attenuates blood-brain barrier disruption in a rat model of transient focal cerebral ischemia: The roles of hdac and mmp-9 inhibition. J. Cereb. Blood Flow Metab..

[43] Zhang H., Park J.H., Maharjan S., Park J.A., Choi K.S., Park H., Jeong Y., Ahn J.H., Kim I.H., Lee J.C. (2017). Sac-1004, a vascular leakage blocker, reduces cerebral ischemia-reperfusion injury by suppressing blood-brain barrier disruption and inflammation. J. Neuroinflam..

[44] Seo W.J., Ahn J.H., Lee T.K., Kim B., Lee J.C., Park J.H., Yoo Y.H., Shin M.C., Cho J.H., Won M.H. (2020). High fat diet accelerates and exacerbates microgliosis and neuronal damage/death in the somatosensory cortex after transient forebrain ischemia in gerbils. Lab. Anim. Res..

[45] Lee T.K., Hong J., Lee J.W., Kim S.S., Sim H., Lee J.C., Kim D.W., Lim S.S., Kang I.J., Won M.H. (2021). Ischemia-induced cognitive impairment is improved via remyelination and restoration of synaptic density in the hippocampus after treatment with cog-up((r)) in a gerbil model of ischemic stroke. Vet. Sci..

[46] Sharma S.S., Dhar A., Kaundal R.K. (2007). Fetpps protects against global cerebral ischemic-reperfusion injury in gerbils. Pharmacol. Res..

[47] Ahn J.H., Chen B.H., Park J.H., Shin B.N., Lee T.K., Cho J.H., Lee J.C., Park J.R., Yang S.R., Ryoo S. (2018). Early iv-injected human dermis-derived mesenchymal stem cells after transient global cerebral ischemia do not pass through damaged blood-brain barrier. J. Tissue Eng. Regen. Med..

